# Psychological intervention at a primary health care center: predictors of success

**DOI:** 10.1186/s12875-019-1005-9

**Published:** 2019-08-17

**Authors:** Miguel Ricou, Sílvia Marina, Paula Marinho Vieira, Ivone Duarte, Inês Sampaio, Joana Regalado, Catarina Canário

**Affiliations:** 1 Department of Community Medicine, Information and Health Decision Sciences, Faculty of Medicine, Porto University, and CINTESIS, Porto, Portugal; 2 University Institute of Maia, Maia, Portugal; 3 Center for Psychology at University of Porto, Faculty of Psychology and Education Sciences, University of Porto, Porto, Portugal; 4 Trás-os-Montes and Alto Douro University, Vila Real, Portugal; 5 Portucalense University, Porto, Portugal

**Keywords:** Predictors of success, Primary health care, Psychological intervention, Retrospective study

## Abstract

**Background:**

Few studies in Portugal have attempted to assess the impact of psychological interventions in primary health care regarding the problems shared by clients, and which variables predicted the success of this intervention. The current study, therefore, aimed to identify predictors of success related to psychological intervention in a single primary health care center in the north of Portugal.

**Method:**

This was a retrospective study from secondary data, using the data from 1024 clients who attended the psychological consultation at a primary health care center over a period of 8 years. The success of the psychological consultation was defined according to the discharge made by the psychologist. The multiple logistic regression analysis was employed.

**Results:**

The attendance of a greater number of consultations and the biweekly frequency of consultations significantly predicted the success of psychological intervention. Additionally, the success was associated with having a diagnosis or specific problem identified.

**Conclusions:**

These findings provide contributions to enrich the literature in this field, in particular, in Portuguese primary health care. We highlight the importance of investing in psychological services in primary health care centers.

## Background

Psychological intervention is defined as a relationship aimed at promoting a better adaptation of the individual to a given situation and thereby optimizing his or her personal resources in relation to autonomy, self-knowledge and self-help [1]. In other words, psychological intervention aims to produce a personal change leading to higher functional results. In the context of primary health care, psychological intervention is characterized by a set of competencies that involve those related to the individual intervention as well as those related to the areas of health promotion and disease prevention, long-term care, humanization, quality, research and training [2]. In this regard, we used a cognitive behavioral intervention. The most widely used therapy in primary health care is cognitive behavioral due to its low cost and high effectiveness [3–5]. The precise number of sessions of cognitive behavioral therapy differs according to the psychological problem; for example, for anxiety the recommendation is at least 5 sessions, while depression is from 6 to 8 [6]. Cognitive behavioral theory has also demonstrated its effectiveness with children [7] through ludic activities [8].

In the most countries of the world, including developing countries, mental health disorders are not treated with the same importance as physical health [9]. According to the world health organization, mental health comprises perceived self-efficacy, subjective well-being, autonomy, self-achievement of intellectual and emotional potential, among others. However, the most common mental health disorders on primary health care setting around the world are depression (vary between 5 and 20%), anxiety (4 to 15%), alcohol addiction (5 to 15%) and somatization disorders (0.5 to 11%) [9]. The utmost usual method of treatment to these mental health disorders is psychotropic medication [6]. Possibly, this situation arises due to the reduced accessibility to psychological interventions.

In Portugal the reality is not different. It is estimated that 1 in 5 Portuguese citizens suffer from a psychological health problem, becoming the second European country with a higher prevalence of psychological health problems [10, 11]. The most common psychological health problems are related to anxiety disorders (an annual prevalence of 16.5%) and mood disorders (an annual prevalence of 7.9%) such as depression [10]. Within this framework, the investment in mental health services in the primary health care system is of utmost importance.

Primary health care is the first-line services and the psychological service it is integrated into the health care centres. Psychological interventions are carried out by psychologists, members of the Portuguese Order of Psychologists. The referrals for the psychological consultation are made by the general practitioners doctors and that is a requirement from Portugal national health system. In agreement with the stated above, cognitive behavioral therapy is one of the frequently used psychological intervention models in Portuguese primary health care [3–5] and it can be designed as a brief intervention technique [5], since as the average number of consultations is ranged between 5 and 8.

Intrinsic motivation has an important role in the success of psychological interventions [12, 13], as well as the therapeutic alliance [14–16]. In addition, the problem needs to be clearly defined to promote a better therapeutic alliance [17, 18].

A higher frequency of sessions seems to contribute to the success of psychological consultations in all age groups [19, 20], although in problems considered severe this effect is rather less well defined [16]. Others predictors of success are being female [21, 22], having a higher number of consultations [4, 5, 23], being older [24–28] and being more motivated to change [13].

Considering the previous findings regarding to the predictors of success of psychological intervention in primary care, we intend to improve knowledge of the factors that contribute to this success in Portuguese primary health care services. In this study the success of the psychological consultation was defined through the number of cases that ended the psychological intervention and discharged by the psychologist, being a decision making between the psychologist and the client (We use the term client instead the term patient. In psychology the term client is traditionally used to refer to the patient, thus distinguish itself from the association with physical illness). when they agree that the goals established where achieved. We also intend to study the multivariate effect of diagnoses according to the age and number of consultations and evaluate the association between diagnosis and the dropout rate of psychological consultations.

## Methods

### Participants

The study’s sample is composed of 1024 clients enrolled in psychological consultation of a primary health care center of the north of Portugal. To be included in the study sample clients must to have attend the psychological service. No exclusion criteria were applied. The age of the study participants ranged between 1 and 88 years (*M* = 28.3, *DP* = 19.3), lower than the average age of the Portuguese population which is 43.9 years old [29]. A majority of the sample was female (*n* = 640, 63%) while 37% (*n* = 375) was male. Most clients (90%, *n* = 882) were referred to the psychological consultation by their *general practitioner doctor* (GP) that traditionally play the role of gatekeeper [29] in the primary health care system.

### Materials and procedure

The current study was a retrospective, and developed from secondary data, using the data from 1024 clients who attended the psychological consultation at a primary health care center, over a period of 8 years, from 2006 to 2014. The data was collected from the HIGIA medical software (v7.1), a health management software that allows health professionals to manage information regarding client’s consultations and clinical data. At the time of the psychological intervention at the health care center and data collection, this was a popular software in Portuguese primary health care centers. In the health care center where data were collected the software was not an online system, and data were stored in a server located in the health care center building.

Diagnosis were established according to the DSM-IV-TR [30]. However, due to the large variety of disorders founded, the diagnoses were grouped in categories according to the main diagnostic class (developmental disorder, mood disorder, anxiety disorder, relational problem, socio-economic problem, other problems, and no diagnosis or condition).

It was considered a success of the psychological consultation, the number of cases that ended the psychological intervention with discharge by the psychologist. This discharge it’s a common decision between the psychologist and the client when they agree that the goals established in the beginning of the process where achieved.

All necessary ethical procedures were fulfilled and assured the anonymity of the data. The study was approved by the ethics committee of the primary health care center where it was held.

### Data analysis

The statistical analysis was performed using the *Statistical Package for the Social Sciences* (IBM SPSS, version 22.0). To evaluate the success of the psychological consultation, a logistic regression analysis (forced entry method) was performed where the client discharge made by the psychologist was included as the outcome variable and the number and the frequency of consultations, the average time of psychological follow-up and the age of clients were included as predictors. To determine whether clients’ age, number of consultations, and average follow-up time differed according to the diagnosis, a multivariate analysis of variance (MANOVA) was performed including the diagnosis as the independent variable and the clients age and the consultations number as outcome variables. And, to better understand associations between diagnosis, gender, and consultation success an association chi-square test was performed.

## Results

### Descriptive results

As presented in Table 1 diagnoses were grouped in the following categories: a total of 15.9% of the clients were coded with relational disorder, 11.5% developmental disorders, 10.2% socioeconomic problems, 10.1% anxiety disorders, 6.3% mood disorders, and 16.6% of the clients were coded with other problems such as academic, religious or spiritual problems, occupational or accommodation problems. Finally, 14.5% other clinical situations that did not fit on a precise diagnosis. Data regarding 14.8% of participants was missing regarding diagnosis.

**Table 1:** Frequencies of the clients’ demographic and consultation characteristics

	Number	Percent
Gender		
Male	375	31.2
Female	649	54.0
Age groups		
10 or less	241	20.0
11 to 20	187	15.6
21 to 30	176	14.6
31 to 40	158	13.1
41 to 50	105	8.7
51 or more	157	15.3
Diagnosis		
Relational disorder	191	15.9
Developmental disorder	138	11.5
Socioeconomic problems	123	10.2
Anxiety disorders	122	10.1
Mood disorders	76	6.3
Other problems	200	16.6
No diagnosis	174	14.5
Consultation status at data collection		
Discharge	330	27.5
Ongoing	132	11.0
Drop-out	293	24.4
Referral to other professionals	83	6.9

Almost half of the psychological consultations had a biweekly frequency (48.6%). The number of consultations per client ranged between 1 and 75 (*M* = 7.30, *SD* = 8.80) and the psychological follow-up were ranging from 1 to 61 months (*M* = 4.70, *SD* = 6.90).

At the time of data collection, 330 (27.5%) clients had successfully completed the psychological intervention process. One hundred and thirty-two clients (11.0%) were still attending psychological consultation, 24.4% represented the dropout rate and 6.9% were referred to another health professional, such as couple therapists, psychiatrists in the case of adult clients or child psychiatrists in the case of the younger clients. 24.4% of the clients returned to the consultation after being discharged. 21.5% of those clients had a problem associated with the previous diagnosis, whereas the remaining 79% came back because other different problems (such as grief by the recent loss of a family member, or work-related problems that were not the reason why they first were followed at psychological consultation).

### Predictors of success

The multiple logistic regression analysis was employed to conduct predictive models of psychological success, considering client discharge (*n* = 330). The number of psychological consultations and the frequency of the consultations were found as predictors of success for the psychological intervention. As presented in Table 2, the regression model evaluated in the logistic regression analysis was statistically significant the number and frequency of consultations were identified as predictors of success, whereas clients age and the average time of psychological follow-up were not. Thus, having a greater number of consultations and a in a biweekly frequency were predictors of success of psychological intervention.

**Table 2:** Predictors of success of psychological consultation

Variable	Model			*CI 95%*
	*Β (SE)*	Wald	*OR*	
Constant	1.61 (.61)	7.05	4.99**	
Age	0.01 (.01)	1.88	1.01	1.00–1.02
Number of consultations	−0.15 (.03)	18.29	.86***	0.81–0.92
Frequency of consultations	−0.62 (.27)	5.35	.54*	0.32–0.91
Average time of psychological follow-up	0.002 (.04)	0.002	1.00	0.93–1.08

Note. *χ*^*2*^ (4) = 67.69, *p* < .001, Cox & Snell R Square = .14, Nagelkerke R Square = .19, *p < .05 **p < .01 ***p < .001

### Multivariate effect of the diagnosis on age and number of consults

To determine whether clients’ age and number of consultations differed according to the diagnosis, a multivariate analysis of variance (MANOVA) was performed including the diagnosis as the independent variable and the clients’ age, consultations number, and average follow-up time as outcome variables (M = 1008). Results revealed the multivariate effect of [*Wilk’s Lambda* = .74, *F* (18, 2826.08) = 17.33, *p* < .001]. The advantage of the multivariate analysis is that it possible to separate the effects of the factors.

Diagnosis had an effect on age [*F* (6, 1007) = 42.72, *p* < .001], on the number of consultations [*F* (6, 1007) = 11.04, *p* < .001], and on the average follow-up time [*F* (6, 1007) = 10.32, *p* < .001] (Table 3). Scheffe’s Post Hoc tests, allowed performing multiple comparisons revealing that: clients with developmental disorders had the youngest ages (*ps* < .001), whereas clients with mood disorders had the oldest ages (*ps* < .001); clients with developmental, mood or anxiety disorders had more consultations than those with relational, socioeconomic or no diagnosis (*ps* < .001); and clients with developmental, mood and anxiety disorders, or other problems were followed for longer periods of time when compared to those with no diagnosis (*ps* < .001).

**Table 3:** Descriptive statistics and univariate and effects of clients’ diagnosis on age, number of consultations and average follow-up time

Diagnosis	Age M (SD)	Number of consultations M (SD)	Number of consultations min-max	follow-up in month M (SD)	follow-up in month min-max
Developmental disorders	9.3 (6.1)	9.93 (9.2)	1–57	6.1 (7.1)	0–38
Mood disorders	44.8 (16.6)	8.6 (8.9)	1–37	6.0 (6.9)	0–31
Anxiety disorders	29.9 (15.5)	9.8 (12.2)	1–75	6.5 (8.3)	0–47
Relational problems	27.7 (20.7)	7.6 (7.1)	1–42	5.2 (6.1)	0–39
Socioeconomic problems	33.2 (21.3)	6.1 (5.4)	1–30	3.9 (4.8)	0–36
Other problems	32.5 (15.9)	7.7 (10.2)	1–59	5.0 (7.9)	0–61
No diagnosis	27.4 (19.0)	3.2 (5.6)	1–59	1.4 (3.7)	0–46
F(6, 1007)	42.72***	11.04***		10.32***	

*Note.* Multivariate effects *Wilk’s Lambda* = .74, *F* (18, 2826.08) = 17.33, *p* < .001, ***p < .001

### Associations between gender and diagnosis and the success of psychological consultation

Consultation success (*n* = 330, discharge) was associated with clients having a specific diagnosis or problem (developmental, mood or anxiety disorder, relational problems or socioeconomic problems) rather than not having a specific diagnosis or problem (*χ2* (6) =34.84, *p* < .001). Likewise, as presented in Table 4, consultation success was not associated with clients’ gender.

**Table 4:** Associations between gender and diagnosis and the success of psychological consultation

	Consultation success		Tests of association
	Yes (%)	No (%)	
Gender			Fisher’s exact test = .80
Female	53.5	46.5	
Male	52.2	47.8	
Diagnosis			*χ2* (6) =34.84, *p* < .001
Developmental disorder	57.3	42.7	
Mood disorder	41.8	58.2	
Anxiety disorder	50.6	49.4	
Relational problems	64.1	35.9	
Socioeconomic problems	66.7	33.3	
Other problems	64.1	56.9	
No diagnosis	34.6	65.4	

*** *p* < .001

## Discussion

The current study results pointed out that the attendance of a greater number of consultations and the biweekly frequency of consultations significantly predict the success of psychological intervention. Additionally, the success was associated with having a diagnosis or specific problem identified. Results also revealed that in clients with developmental disorders as the youngest, and clients with mood disorders as the oldest, clients with developmental, mood or anxiety disorders had more consultations, and those with no diagnosis presented the least follow-up time.

The sample of this study (*N* = 1024) corresponds to the total number of clients enrolled in psychology consultations in the health care center in which the study took place, over a period of 8 years. The average age of the sample is about 28 years, lower than the average age of the Portuguese population, which is 43.9 years old [31]. The lower average age of this study can be explained by the high number (34.7%) of children (up to 15 years) included in the sample.

Developmental disorders represent 11.5% of the diagnoses of this study. These data are related to the high number of children in the study sample. The results may also indicate that the Primary Health Care Units (PHCU) are required by the parents of children with these types of disorders; this may be because it is easier for the parents or because few options for psychological intervention are available in other contexts such as schools or hospitals.

Most of the participants in the current study were referred to psychological consultations by their GP. These professionals have traditionally taken on the role of *gatekeeper* [29] of PHCU, and are responsible for guiding clients through the national health service. This is why it is the GPs who are largely responsible for referring clients to psychologists. Furthermore and due to the organization of the Portuguese health system, it was not possible for a client to make an initial appointment with the psychologist without first seeing the GP, except with a direct indication from the psychologist. Such a situation could be a way of understanding the diagnoses found in this sample. Most of the cases identified are linked with problems of daily life (i.e., academic, professional or religious or spiritual problems). These problems are usually less valued from a medical point of view, and so these clients are usually referred for psychology consultations. These diagnoses will likely not be representative of their prevalence in the Portuguese population but will be influenced by the context of intervention in health care centers. In fact, people do not go to the hospital or private clinics for non-clinical reasons; the health center and the family doctor, therefore, are the more logical option. The next logical step is to forward the clients without a clear clinical diagnosis for psychology consultations. Thus, we can better understand these results; namely, the existence of 43% of cases with daily life problems, of which 17% are related to experiential issues (bereavement, academic, professional and religious or spiritual problems, among others), 16% relational disorders and 10% related to socioeconomic conditions. On the other hand, there is a prevalence of 6% of mood disorders and 10% of anxiety disorders. Anxiety and mood disorders are indicated as the most common psychological health problems around the world including Portugal [6, 9, 10], which goes against the obtained results. However, taking into account that the northern region of Portugal is the region with the highest rate of unemployment [31], these results can be justified in part. On the other hand, it is possible that GPs can adopt a pharmacological intervention in clients with clear clinical characteristics, such as mood and anxiety disorders, referring the clients without a clear clinical significance for psychological consultation. In Portugal the benzodiazepines consumption was studied, and an excessive consumption of this psychopharmacological drugs was found [32].

It is important to note that in 15% of cases, it was not possible to accurately classify the problem. In fact, this may be due to difficulty defining the request of the clients, thus making it more difficult to diagnose the problem. Clients are often referred for psychological consultation by their GP, and they may have no other motivations or conscience about their problems. They come to the psychologist just because the doctor said to do so. The definition of the diagnosis can be important in the success of the psychological intervention. We will back to this topic later on the discussion about the association between consultations discharge by the psychologist and the diagnosis.

The main objective of this research was to ascertain the predictive factors of the success of psychological interventions in this PHCU.

In the literature [24–28], age seems to be a predictor of success of psychological intervention. In this study, there was a positive association between the age and the diagnosis of the subjects of this study sample. Developmental disorders are most predominant in younger people. In turn, in older people there is a higher frequency of mood disorders. Children are developing and growing, so they have several peculiar susceptibilities that adults do not have [28]. In turn, older people are logically in a more advanced stage of life; they have experienced more significant losses and are more prone to economic difficulties and isolation [24]. In addition, this age group is more prone to a number of serious and limiting diseases, increasing the use of pharmacological drugs that may predispose or worsen possible symptoms of depression [24]. Older people have a particular set of characteristics conducive to the development of mood disorders [25]. In a study conducted by Target and Fonagy (1994), it was indicated that children under 12 years of age demonstrate greater changes than older children. Moreover, when compared to adults, they are less influenced by previous assumptions and pay more attention to current evidence [26]. With the advancement of age, learning requires a greater effort to be effective, meaning that the capacity to acquire new skills reduces [27]. Within this framework, younger age can constitute a predictor of success for psychological interventions.

For psychological interventions to be effective, an adequate number of psychological consultations must be performed [33]. According to Craske and collaborators and as verified in this study, a greater number of consultations indicates a greater probability of success in the intervention process. Cognitive behavioral therapy has been one of the most widely studied psychological intervention models [3] and it is frequently used in PHCU due to its low cost and high rate of effectiveness [4, 5]. The average number of consultations considered ideal is between 5 and 8, and it can therefore be designed to be a brief intervention technique [5, 23]. These data are in agreement with the present study in which the mean number of consultations is approximately 7.

In contrast, an increase in dropout rates is significantly associated with a lower number of consultations [20]. In fact, in this study, it was verified that about 35% of client’s dropout before their second consultation and, of those who continued, only about 19% dropout before their final psychological consultation. Initially, there is no effective therapeutic bond between psychologist and client. The therapeutic alliance is very important during psychological interventions, but takes time to develop [14]. On the other hand, it may be that clients, after their first consultation, feel better and cease the psychological follow-up [16]. In fact, there are different stages for establishing the therapeutic alliance. Of course, the first consultation is an important moment. However, it will be during the intervention that the psychologist can promote an environment in which the client feels understood and connected with their psychologist. As the process evolves, change becomes more probable [15]. The best results occur when the client becomes actively involved in the process and personally invests in the change [34].

The biweekly frequency of consultations proved to be a predictor of success in this PHCU. In fact, considering the high affluence of psychology consultations in the PHCU, only the clients evaluated by the psychologists as more severe had consultations on a weekly basis. It is not surprising then that biweekly frequency is a predictor of success, not because this frequency is considered ideal in the process of psychological intervention but because the worse prognoses are filtered out and followed more frequently [16]. Biweekly frequency arises due to an organizational constraint; in fact, given the high number of requests for psychological consultation, it is impracticable to promote an ideal setting (i.e. a weekly follow-up) [4, 5, 23].

Another finding is the positive association between consultations discharge by the psychologist and the diagnosis. The lack of diagnosis indicates a high number of dropouts; clients who present symptomatology without a specific diagnosis have higher rates of dropout. This result suggests the importance of problem identification by the client in order to increase his or her motivation for psychological intervention. According to the literature [12, 13], motivation is considered to be an excellent predictor of the success of psychological intervention, and demotivation is likely to result in the client abandoning the therapy. The client’s demand for problem identification seems to be an essential element in their motivation during the therapeutic process [17], once it main goal is to increase the client’s self-awareness and their awareness of the problem [18]. Clear identification of the problem is an indicator of success because it is closely related to motivation [13], which in turn supports the success of the overall psychological intervention [17, 18].

In summary, the lower number of consultations in clients without a specific diagnosis justifies their higher dropout rate. A clear definition of the problem seems to be crucial to motivate the client during psychological interventions. We can say then that psychologists should try to improve the definition of the problem with the client, more so in primary health care because clients are usually referred to the psychological consultation by their GP. This way, GPs can refer clients to a psychologist if they are unable to make a clear diagnosis.

Efforts have been made in Portugal [11] and in other countries to restructure mental health services in order to promote more diversified support throughout the population. About 60% of people who use PHCU have a mental disorder that can be diagnosed, and it is clear that the integration of mental health services into the PHCU generates excellent results for the promotion of health at a reasonable cost [9]. At the end of this study, about 35% of participants had successfully completed the psychological intervention; however, there are no similar studies in Portugal for comparison that would allow us to better understand these outcomes. For respond to this lack it could be important the replication of this study at a national level.

Additionally, the ratio of psychologists in primary health care centers should be reconsidered due to the disproportionality between the number of psychologists versus the number of clients. The frequency and number of psychology consultations are far below what the population needs. In fact, due to the high demand for psychological consultations, psychologists had allocated weekly consultations solely to clients who presented more intense symptomatology. For that reason, clients with biweekly consultations had less severe symptoms and so possessed better prognoses [15, 16, 20]. It is important to invest in studies that identify and evaluate factors that can influence the effectiveness of psychological intervention in order to increase the quality of psychological services in primary health care units and, consequently, increase the quality of life of the population. Additionally, investing in the development of consistent mental health policies according to the needs of the population allows the reduction of hospitalization rates and medication costs.

## Conclusions

In conclusion, our findings are in line with the research made about the success of psychological intervention. The number of consultations and a biweekly frequency appears to benefit from greater rates of success in psychological consultations. This study, from a Portuguese sample, identify the success predictors of psychological consultation in Portugal, supporting the international studies on the subject. Additionally, the association between diagnosis and the success of psychological consultation was an important finding in this study. A clear identification of the problem and an adequate referral for the psychology service seems to be important to the success of psychological intervention. Future studies that explore this issue is needed.

## Data Availability

All data generated or analysed during this study are included in this article.

## Abbreviations

GP: General Practitioner
PHCU: Primary Health Care Units
